# Suicide

**Published:** 1897-03

**Authors:** 


### EDITORIAL DEPARTMENT.

F. E. DANIEL, M. D., Editor.
S. E. HUDSON, M. D., Managing Editor.
A. J. SMITH. M. D., Galveston, Associate Editor.

EDITORIAL STAFF:

PROF. J. E. THOMPSON, M. D., Texas Medical College, Galveston; Surgery.
PROF. WM. KEILLER, M. D., Texas Medical College, Galveston; Obstetrics and Gynecology.
PROF. DAVID CERNA, M. D., Texas Medical College, Galveston; Therapeutics.
PROF. A. J. SMITH, M. D., Texas Medical College, Galveston; Medicine.
DR. R. H. L. BIBB, City of Mexico; Foreign Correspondent.

Published Monthly at Austin, Texas, by Drs. Daniel and Hudson. Subscription price $2.00 a year in advance.

Eastern Agents: The Monihan-Fairchild Special Agency, 24 Park Place, New York City.

Official organ of the West Texas Medical Association, the Houston District Medical Association, the Austin District Medical Society, Brazos Valley Medical Association, the Galveston County Medical Society, and several others

### SUICIDE.

The subject of suicide is, more than ever, occupying attention, not only of scholars and writers, but of all classes. The question as to an individual's right to terminate his life,—for reasons satisfactory to himself,—is being discussed in medical, religious and secular journals, and even from the pulpit; and it cannot be denied, whether as a result of this discussion, and the directing of attention to the subjector not,—the idea that one has that right, is growing in popular favor. The number of suicides increases each year.

It may be that with the growing neglect of religious duties, and the consequent removal from the influence of the church, the fear of future punishment is greatly diminished; and this restraint, which has always been the most potent factor in limiting the number of suicides, no longer deters many so disposed. It is this fear, rather than any moral consideration of right or wrong, or of responsibilty, that has operated to hinder the despondent and the unhappy.

And, when one reflects on this phase of the subject; that the dread of the orthodox hell, which at one time made sinners crouch in agony in their church pews, and pray to be delivered, is no longer felt by many, and the hell itself, regarded as a fiction, has become a subject of contemptuous disregard, if not of open ridicule; that many persons no longer *fear* to die,—it is truly remarkable that the number of suicides is not a thousand fold greater than it is. There is more suffering in the world, the consequence, first and foremost, of whisky,—and of poverty,—overcrowding of population, competition in all the channels of human industry; more bitter struggle for the necessaries of life than ever before, and when one realizes the existence of this misery, it seems strange that so many thousands, who are irretrievably doomed to such life, with not a glimmer of hope of betterment, will accept life at such cost.

In New York, recently, Rev. LeRoy Wright, the pastor of one of the popular churches, startled his congregation and the public, created a sensation, by openly advocating and advising suicide as an escape from life when its burdens became intolerable. Who can estimate the extent of the evil influences of such preaching? He says,—suicide is a concomitant of civilization and progress, and that, as civilization advances, suicide will increase in a corresponding ratio. It is interesting to note, in connection with this sermon, that an eminent lawyer of New York has given it as his opinion that the reverend gentleman has laid himself liable to prosecution; for, under the laws of that State, one who "advises, encourages or abets suicide," is *particeps criminis*, and is guilty of a felony. The efforts to prevent suicide by law, have always been absurd and ineffectual.

If this reverend gentleman is right, in the opinion that suicides will increase *pari passu* with civilization, the day will come when it will be a sanitary evil, as well as a moral one. Suicides will become so common, as it grows in favor,—as poverty and disease press on;—as the struggle for bread becomes fiercer;—and when the idea of a hell is held in utter contempt;—when "the proud man's contumely," and "the oppressor's wrongs" become still more unbearable; when the *facilities* increase, afforded by a better knowledge of the means whereby life may be destroyed, and men can make their quietus by means more sure, and less clumsy and revolting than the extemporized halter, or a header in the cold waves of an inhospitable river, or lake, it will be a problem what to do with the corpses!

Indeed, the Rev. Mr. Wright is not alone is his opinion, by any means; many scholars are of his way of thinking. Ignatius Donnelly held the same views, and in a most interesting book, published in 1889 ["Cæsar's Column"], anticipated this sanitary want, and suggests a remedy.

He pictures civilization one hundred years hence—when the rich have become inordinately rich, and the great army of toilers unbearably, intolerably poorer; when, with the acme of perfection in all the arts and sciences, there shall have come the apotheosis, on the one hand, of luxury and indolence, and on the other, the culmination of human suffering, suicide will be so common and frequent that the streams will become polluted by the carcases, and the air, on the highways and byways, will be poisoned by the putrefaction of decaying bodies! To meet and remedy this evil, institutions for the promotion and facility of suicide are opened in every large city, where, by means of publications and free lectures, the public are instructed in what way one may best shuffle off the intolerable "coil;" are made acquainted with the various poisons, and their mode of action; their relative and comparative life-destroying qualities; and are, moreover, supplied with a quantity of each, from which a choice may be made. Every facility is afforded. There are nice, quiet apartments to which the unhappy one may repair, read up on the various routes, select the one he prefers, and thus armed with the means, furnished him by the institution at government expense, he may quietly extinguish the vital spark with neatness and dispatch, and without pain or expense; and in connection with the establishment, there is a crematory, where the sanitary problem above referred to is speedily solved. And there you are.

There is philosophy in this. If one has determined the question for himself, (and unless he has dependencies, no one else should care, or have any voice in the matter); if, we say, one has determined that it is "nobler in the mind" to throw up the sponge, than to endure "the whips and scorns of outrageous fortune," why, let him do it with due regard for the proprieties, and for the rights of others; let it not be a nuisance, or a shock to the living.

Without expressing any opinion on the subject of one's right to terminate a life no longer desirable, we can easily imagine conditions,—thousands of them; and, indeed, we need not *imagine* them; every man who will look around him in any large city will see and realize conditions which even to the most orthodox beleiver in future punishment would be a great temptation, at least, to fly to the ills they know not of, in preference; conditions so tempting as to well nigh outweigh every consideration of right or wrong,—and still the wonder grows that so many will groan beneath the burden of an intolerable life rather than seek the Lethean waters of oblivion.

When science shall have banished both war and pestilence, suicide will become a necessity in the equation of human affairs.

A RIGHTEOUS ACT.—The following bill has been introduced into the Kansas legislature, referred to the Committee on Public Health and Hygiene, and by them reported back with the recommendation that it do pass.

Rape and seduction are notoriously on the increase. Statistics published by this journal, taken from the United States census, show an alarming increase in all kinds of crime, a demonstration that hanging is a failure, if its object is to lessen crime. The law should be repealed as to hanging; it is barbarous and worse than useless. There is reason to believe that emasculation would be a better remedy for certain crimes than hanging, and the Kansas people propose to try it. The editor of the TEXAS MEDICAL JOURNAL has long advocated such a measure, and a few years ago a bill similar to the following was introduced into the Texas legislature, but it got lost in the shower of political matters, and never came to light again.

The following is the text of the Kansas bill. It was formulated, we think, by Dr. F. Hoyt Pilcher, late superintendent of the Institute for Idiots and Feeble Minded, at Winfield, Kans., and who, it will be remembered, castrated six of the inmates of that institution, with splendid results, for the cure of masturbation.

This is known as the Pilcher bill:

AN ACT in relation to crime and punishments, and to provide for the castration of persons who shall be convicted of certain offenses therein named.

Be it enacted by the legislature of the State of Kansas:

Amended by striking out all after the enacting clause and substituting the following:

SECTION 1. Every person who shall hereafter be convicted of rape as such crime is defined by section 2153 of the general statutes of 1889, or who shall forcibly ravish any woman; and every person who shall hereafter be convicted of incest as such crime is defined by section 2367 of the general statutes of 1889; and every minister, clergyman, priest or teacher, having charge of any church or other religious body, or school, who shall have illicit connection with any unmarried virgin female under twenty-one years of age of his charge or school; and every guardian of any female ward under the age of eighteen (18) years who shall defile her, shall be punished by imprisonment at hard labor for a period not less than five nor more than twenty years, and in addition to such punishment, he shall be castrated.

SEC. 2. In all cases where the defendent shall be convicted of any of the offenses specified in the foregoing section, the court shall, when pronouncing judgment upon the defendant, order that the prisoner shall, after the expiration of ore year from the date of his arrival at the penal institution in which he is to be confined, and within eighteen months from the date of the sentence, be castrated by the medical officer of such penal institution; which order shall be a part of the final judgment in such case, and a certified transcript of which order and judgment shall be delivered with the prisoner to the principal officer of the penal institution in which the prisoner is to be confined.

SEC. 3. It shall be the duty of the principal officer of the penal institution having the custody of the person so convicted and sentenced, after one year shall have expired from the time of receiving such prisoner, and within eighteen months from the date of the sentence of the court, to cause the medieal officer in charge of the health of his prisoner to castrate the prisoner under such sentence; and if such medical officer for any cause shall fail to perform such operation, or to cause the same to be performed when required of him, the principal officer in charge shall call in some competent surgeon to take charge of and perform such operation.

SEC. 4. In any case where the order for such operation has been carried into effect under the provisions of this act, and after two months from the time of such operation, the governor may, if satisfied that the prisoner will lead a peaceful and orderly life, issue to such prisoner a parole or conditional pardon, to be valid only during the good behavior of such person.

SEC. 5. All laws and parts of laws in conflict with the provisions of this act are hereby repealed.

SEC. 6. This act shall be in force from and after its publication in the official State paper.

